# GASC1-Adapted Neoadjuvant Chemotherapy for Resectable Esophageal Squamous Cell Carcinoma: A Prospective Clinical Biomarker Trial

**DOI:** 10.1155/2020/1607860

**Published:** 2020-01-30

**Authors:** Ruinuo Jia, Youjia Mi, Xiang Yuan, Dejiu Kong, Wanying Li, Ruonan Li, Bingbing Wang, Yafei Zhu, Jinyu Kong, Zhikun Ma, Na Li, Qiangjian Mi, Shegan Gao

**Affiliations:** ^1^Cancer Hospital, The First Affiliated Hospital of Henan University of Science and Technology, Luoyang, Henan 471000, China; ^2^College of Clinical Medicine of Henan University of Science and Technology, Luoyang, Henan 471000, China; ^3^Julius L. Chambers Biomedical/Biotechnology Research Institute, Department of Biological and Biomedical Sciences, North Carolina Central University, Kannapolis, NC 28081, USA

## Abstract

Neoadjuvant chemotherapy (NCT) is a standard care for esophageal squamous cell carcinoma (ESCC), but the efficacy is unsatisfactory. Cancer stem cells (CSCs) play key roles in chemotherapy resistance. Gene amplified in squamous cell carcinoma 1 (GASC1) is a neoteric gene in stemness maintaining of ESCC. We aimed to reveal whether GASC1 could be a predictive biomarker for NCT in ESCC. ESCC patients (T2-4N0-2M0) were evaluated for GASC1 expression using immunohistochemical staining and classified as GASC1-low group (GLG) and GASC1-high group (GHG). NCT was delivered in two cycles and then the surgery was completed. Primary endpoints were tumor regression grade (TRG) and objective response rate (ORR); secondary endpoints were radical surgical resection (R0) rate and three-year overall survival (OS). 60 patients were eligible with evaluable outcomes: 24 in GHG and 36 in GLG. Between GHG and GLG, TRG1, TRG2, TRG3, and TRG4 were 0 : 16.7%, 20.8% : 41.7%, 58.3% : 36.1%, and 20.8% : 5.6%, respectively (*P*=0.006); ORR and R0 rate were 33.3% : 69.4% (*P*=0.006) and 75% : 94.4% (*P*=0.046), respectively; the median OS was 20 : 32 (months) (*P*=0.0356). No significant difference in the three-year OS was observed between GHG and GLG: 29.2% : 41.7% (*P*=0.24). Furthermore, the GASC1 expression level was associated with poor OS independent of other factors by univariate and multivariate analyses. Therefore, GASC1 might be a potential biomarker to predict NCT efficacy for ESCC.

## 1. Introduction

Esophageal cancer (EC) is the eighth most common cancer worldwide and the sixth leading cause of cancer-related deaths [1]. China is the country with the highest incidence of EC in the world. Contrary to the European and American countries where 80% of EC is adenocarcinoma, more than 90% of EC in China is esophageal squamous cell carcinoma (ESCC) [2]. The neoadjuvant treatment has become the standard of care for patients with resectable EC [3, 4]. However, the efficacy of neoadjuvant chemotherapy (NCT) is unsatisfactory in ESCC, but some patients cannot undergo surgery or curative resection because of disease progression [5]. Markers predicting response to chemotherapy would considerably enhance the efficacy of treatment and simultaneously reduce chemotherapy-related risks by permitting precise preoperative treatment [6–8].

Cancer stem cells (CSCs) are responsible for cancer growth, metastasis, and recurrence and anticancer drug resistance [9–12]. Therefore, the genes that are involved in CSCs maintenance could be the potential predicting biomarkers for precision ESCC chemotherapy.

The gene amplified in squamous cell carcinoma 1 (GASC1, also named KDM4C/JMJD2C), which encodes a nuclear protein with a Jumonji C domain that catalyzes lysine (K) demethylation of histones, is essential to maintain the self-renewal and differentiation of embryonic stem cells [13]. In previous studies, GASC1 was shown to be a regulator of stemness in CSCs of ESCC via pluripotency-associated genes (PAGs) promoter demethylation, such as NOTCH1 and SOX2 [14, 15], and the high level of GASC1 was closely associated with poor survival of ESCC patients [14–16].

As a result, we prospectively investigated the role of GASC1 as a biomarker to predict the efficacy of NCT for ESCC. We found that a high level of GASC1 was closely associated with worse response to NCT and poor survival of ESCC patients, which could provide a theoretical basis for the development of a new therapeutic strategy of ESCC.

## 2. Materials and Methods

### 2.1. Study Design

**Figure 1:**
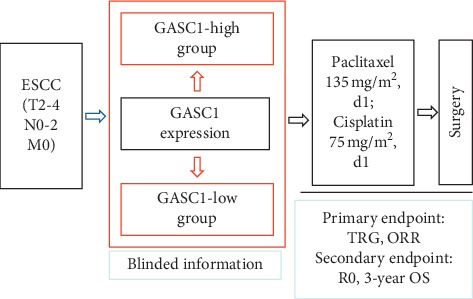
Study flow chart.

This study was a prospective clinical biomarker trial and designed to demonstrate the predictive role of GASC1 for indicating ESCC patients to receive neoadjuvant chemotherapy (Figure 1).

### 2.2. Participants

The target population was locally advanced ESCC, defined as pathologically proven ESCC, with clinical stages of TNM classification, T1bN + M0 and T2-4aN0-2M0, according to the American Joint Committee on Cancer Staging System (UICC-AJCC) (8th edition) [17]. All participants were enrolled from The First Affiliated Hospital of Henan University of Science and Technology (HUST), Luoyang, China.

### 2.3. GASC1 Detection

Immunohistochemical staining was conducted according to the procedures described previously [15]. The sections were incubated overnight at 4°C with primary antibodies against anti-GASC1 (Abcam, Cambridge, MA, USA). After washing with phosphate buffer saline (PBS), sections were incubated with an appropriate biotinylated secondary antibody (Zymed Laboratories, San Francisco, CA, USA) for 30 min. The primary antibody was replaced with PBS for use as a negative control.

Staining intensity was graded as 0 (no staining), 1 (weak staining), 2 (moderate staining), or 3 (strong staining). The analysis of staining in the normal epithelium showed predominant absence or mild staining. In ESCC samples, grades 0 to 1 were classified as low expression, and grades 2 to 3 as high expression. Scoring was conducted independently by two independent pathologists. Participants were divided into two groups: the GASC1-high group (GHG) and the GASC1-low group (GLG) according to the detection result.

### 2.4. Pretreatment Investigation

According to the 8th UICC-AJCC TNM staging manual, patients received staging workup using esophagoscopy and biopsy, endoscopic ultrasonography (EUS), and CT of the thorax and abdomen with contrast, and ultrasonography of the cervical region with fine-needle aspiration cytology for any suspicious nodes [17]. Positron emission tomography-computed tomography (PET-CT) was used when the disease stage was difficult to confirm by general imaging examination.

### 2.5. Interventions

Patients received protocol-defined therapy in 2 stages: neoadjuvant chemotherapy and standard esophagectomy.

### 2.6. Neoadjuvant Chemotherapy

The chemotherapy involved a two-cycle TP (paclitaxel + cis-platinum) regimen: both paclitaxel with 135 mg/m^2^ (1 h intravenous infusion) and cisplatin with 75 mg/m^2^ (3 h intravenous infusion) were administered on day 1, with 21 days as one cycle. This regimen was based on the Esophageal Cancer Management Guideline, National Health and Family Planning Commission of the People's Republic of China.

### 2.7. Standard Esophagectomy

Standard esophagectomy surgery was performed for patients by specialists. The surgical approach to the mid- or lower-thoracic esophagus was standardized to two-stage esophagectomy to achieve a 5 cm minimum proximal margin. For tumors located over the proximal mid-thoracic esophagus, where a 5 cm proximal margin could not be achieved, a three-stage esophagectomy was performed. We performed a two-field lymphadenectomy in situations of either cervical or thoracic anastomosis. All the esophagectomies were performed through a thoracoscopy operation or an open approach.

### 2.8. Follow-Up

After the completion of treatment, all patients were regularly invited to follow-up examinations according to the protocol stipulations. The first follow-up was scheduled for about 4–8 weeks after treatment and then in 3 months for three years including clinical examination, gastroscopy biopsy, CT, and ultrasonography.

### 2.9. Endpoints

The primary endpoints were TRG and ORR between the two groups. The secondary endpoint was R0 rate and OS.

### 2.10. Statistical Analysis

This was a biomarker exploratory trial. The sample size required to meet the statistical power is unknown. TP53 was reported as an ESCC biomarker for neoadjuvant chemotherapy based on a 36-patient study [18]. GASC1 was assumed to be less powerful than TP53 for ESCC in this study. 72 (2 × 36 patients) patients were expected to enroll and a 10% missing rate was considered. Therefore, 80 patients were recruited in the initial phase. The endpoints were analyzed only for those who finished both neoadjuvant chemotherapy and surgery.

All statistical analyses were performed using SPSS 18, Inc., Chicago, IL, USA, and data were expressed as mean values ± standard deviation. Two independent samples were compared using a *t*-test. Fisher's exact test or Pearson's Chi-squared test was performed for the categorical variables between groups. Kaplan–Meier curves were analyzed to determine patient survival with the log-rank test to ascertain significance. Significant predictors were assessed by multivariate analysis using the Cox proportional hazards model. All confidence intervals (CIs) were stated at 95%. A two-tailed *P* value less than 0.05 was considered statistically significant.

## 3. Results and Discussion

### 3.1. Patients Characteristics

**Figure 2:**
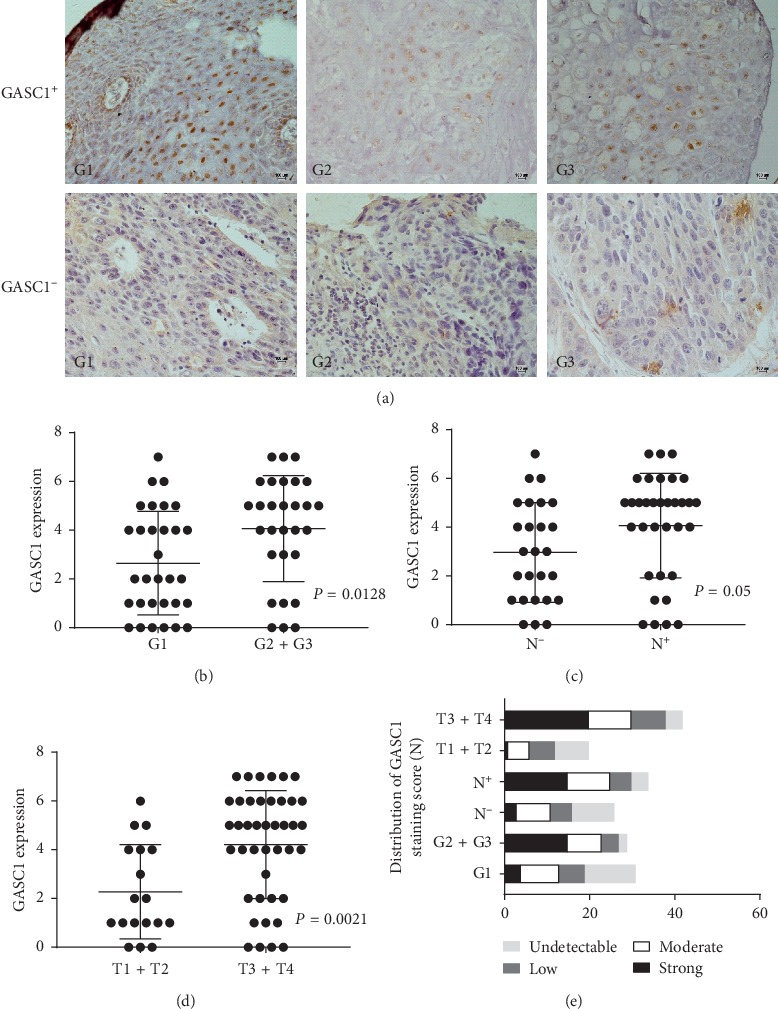
The correlation between GASC1 level and clinical parameters in ESCC patients. GASC1 expression in all ESCC tissues was measured by immunohistochemistry. (a) The expression of GASC1 in different grade tumor tissues from ESCC patients was detected. One representative micrograph is shown. Scale bar represents 20 *μ*m. (b) The expression of GASC1 in different grade tissues (G1, G2 + G3) from ESCC patients is presented as a scatter diagram. (c) GASC1 expression in ESCC tissues with positive and negative lymph node metastasis is shown as a scatter diagram. (d) GASC1 expression in different tumor tissues based upon T score (T1 + T2, T3 + T4) is shown as a scatter diagram. (e) GASC1 expression in ESCC tissues with different clinical parameters analyzed by immunohistochemistry is shown as a histogram with a staining score.

We accrued 80 patients from The First Affiliated Hospital of Henan University of Science and Technology (HUST) from October 2014 to December 2015, of which 60 were eligible with explicit GASC1 expression status and therapeutic evaluation: 24 in the GASC1-high group (GHG) and 36 in the GASC1-low group (GLG). The other 20 patients were excluded from the final analysis since they did not process the surgery and the tissue samples were absence: 1 patient died from pulmonary embolism after 1 cycle chemotherapy; 2 patients gave up surgery and pursued 6-cycle chemotherapy in total, and 17 patients shifted to the definitive concurrent radiochemotherapy when they finished 2-cycle TP regimen. The clinical characteristics of the participants are summarized in Table 1. GASC1 expression status is shown in Figure 2.

### 3.2. TRG, ORR, and R0 Evaluations

After NCT is completed, we evaluated the objective response rate (ORR) depending on the tumor size change in the computed tomography (CT) scan. The modified RECIST (response evaluation criteria in solid tumors) 1.1. was used to define the tumor response: complete response (CR) means that no tumor lesions were seen on the CT imaging; partial response (PR) means regression of the primary tumor and/or lymph nodes; stable disease (SD) means no difference in tumor and/or lymph node size; progressive disease (PD) means progression in size of the primary tumor and/or lymph nodes or development of new lesions [19]. The post-neoadjuvant therapy (ypTNM) stage was depended on the pathological review of surgical specimens [17]. Tumor regression grade (TRG) was quantitated in five grades: TRG 1 (complete regression) showed the absence of residual cancer and fibrosis extending through the different layers of the esophageal wall; TRG 2 was characterized by the presence of rare residual cancer cells scattered through the fibrosis; TRG 3 was characterized by an increase in the number of residual cancer cells, but fibrosis still predominated; TRG 4 showed residual cancer outgrowing fibrosis; TRG 5 was characterized by absence of regressive changes [20]. ORR was defined as CR + PR. A radical surgical resection was defined as follows: R0 means no cancer at resection margins, R1 means microscopic residual cancer, and R2 means macroscopic residual cancer or M1 [21].

TRG1, TRG2, TRG3, and TRG4 between GHG and GLG were 0 : 16.7%, 20.8% :  41.7%, 58.3% : 36.1%, and 20.8% : 5.6%, respectively (*P*=0.006). ORR and R0 rate in GHG were significantly lower than those in GLG: 33.3% versus 69.4% (*P*=0.006) and 75% versus 94.4% (*P*=0.046) (Table 2).

### 3.3. Survival

**Figure 3:**
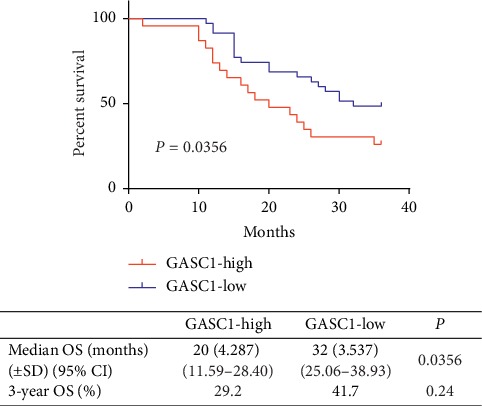
Kaplan-Meier survival curves for ESCC patients with lower and higher GASC1 expressions.

The median overall survival (OS) was 20 months in GHG and 32 months in GLG (*P*=0.0356). The three-year OS rate in GHG was lower than that in GLG: 29.2% versus 41.7%, respectively, but the difference was not significant (*P*=0.24) (Figure 3).

In the univariate analysis, patients with high GASC1 expression levels showed significantly worse OS than those with GASC1 expression levels. Tumor depth, lymph node metastasis, and pathological grade were also significant prognostic factors. In the multivariate analysis, the high GASC1 expression level was significantly associated with poor survival (*P*=0.048) (Table 3).

## 4. Discussion

GASC1, a family number of KDM4 and also named KDM4C, was identified and cloned from the 9p24-amplified region of esophageal cancer cell lines [13]. Histone lysine demethylases (KDMs) regulate histone methylation dynamics and play critical roles in modulating chromatin architecture, gene transcription, and cellular differentiation [21–23]. Dysregulation of KDM4 demethylases has been documented in a variety of cancers, including lymphoma, medulloblastoma, breast, prostate, colorectal, lung, gastric, esophageal, and renal cancers [13, 24–26]. In our previous investigation, we found that GASC1 plays an important role in maintaining ESCC stem cells and participates in tumor development. The expression of GASC1 in a number of ESCC cell lines and poorly differentiated ESCC tissues was higher than that in human immortalized normal esophageal epithelial cell lines and well-differentiated tissues [14, 15]. We identified tumor initial cells (TICs) from a number of ESCC cell lines and demonstrated increased expression of GASC1 in the ESCC ALDH + TICs and its involvement in TICs maintenance by specific demethylation of H3K9Me3 at the SOX2 and NOTCH1 promoter.

Stemness in various types of cancer is the main cause of tumor recurrence, deterioration, and chemoradiotherapy resistance [9–12]. Based on this and according to our previous findings, we assumed that ESCC patients with high GASC1 expression in tumor tissue would respond poorly to neoadjuvant chemotherapy in this study.

The investigation results showed that 40% of patients (24/60) had high GASC1 expression. Additionally, the proportions of lymphatic metastasis, III-IV stages, and pathology grades 2-3 in GHG were significantly higher than those in the GLG: 70.8% versus 38.9%, 93.3% versus 38.9%, and 83.4% versus 55.5%, respectively. This is consistent with the previously published findings [13–16]. After neoadjuvant chemotherapy, six patients achieved TRG1 in the GASC1-low group but none in the GASC1-high group and the ORR was 69.4% (25/36) in the GASC1-low group and 33.3% (8/24) in the GASC1-high group, which indicate that patients with high GASC1 expression would respond poorly to chemotherapy. We found the median OS in the GASC1-high group was significantly worse than that in the GASC1-low group: 20 months versus 32 months, respectively, and GASC1 was an independent prognostic factor for poor overall survival in ESCC patients. GASC1 overexpression proved to predict poor prognosis for some other cancers [24–26].

According to these findings, the potential biological mechanism of chemotherapy resistance is high GASC1 expression enhancing the proportion and ability of the TICs subpopulation in ESCC. However, more exploration into the molecular mechanism is required to confirm this speculation. We have conducted some pilot experiments in ESCC cell lines and mice. The preliminary results support the role of both GASC1 and TICs in ESCC chemotherapy resistance.

We previously reported that caffeic acid (3,4-dihydroxycinnamic acid, CA) could inhibit the demethylation activity of GASC1 in ESCC, and GASC1 was found to confer stem-cell-like characteristics, such as the ability to form spheres, in ESCC TICs [14, 15]. The investigation results provide an innovative concept that GASC1 might be a malignancy signature for ESCC, and CA, a valid GASC1 demethylase inhibitor, could be a potential anticancer agent for ESCC through targeting the TICs. CA has been approved for thrombocytopenia treatment in China by the China Food and Drug Administration (CFDA) [27, 28]. Therefore, we are developing a prospective randomized, double-blind, and multicenter clinical trial called “The Efficacy and Safety of Caffeic Acid for Esophageal Cancer (CAEC)” and patient recruitment is ongoing. The trial has been registered with ClinicalTrials.govIdentifier: NCT03070262.

## 5. Conclusions

In conclusion, our findings firstly indicate that GASC1 is a potential biomarker indicating ESCC patients to receive neoadjuvant chemotherapy. High GASC1 expression could be a prognostic factor for poor survival of ESCC. Our findings provide a theoretical basis for developing a new therapeutic strategy for ESCC based on the inhibition of the GASC1 signaling pathway.

## Figures and Tables

**Table 1:** Patient characteristics in baseline.

Characteristics	GASC1			*P* ^*∗*^
	High (*n* = 24, %)	Low (*n* = 36, %)	Total (*n* = 60)	
Age (year)				
High (≥65)	5 (20.8)	15 (41.7)	20	0.08
Low (<65)	19 (79.2)	21 (58.3)	40	
Gender				
Male	13 (54.2)	24 (66.7)	37	0.24
Female	11 (45.8)	12 (33.3)	23	
Tumor location				
Upper-esophagus	5 (20.8)	6 (16.7)	11	0.731
Middle-esophagus	12 (50)	16 (44.4)	28	
Lower-esophagus	7 (29.2)	14 (38.9)	21	
cT				
T1b	2 (8.3)	8 (22.2)	10	0.16
T2	2 (8.3)	12 (33.3)	14	
T3	10 (41.2)	10 (27.8)	20	
T4a	10 (41.2)	6 (16.7)	16	
cN				
N0	7 (29.2)	22 (61.1)	29	0.015
N^+^	17 (70.8)	14 (38.9)	31	
Clinical stage				
II	4 (16.7)	22 (61.1)	26	0.001
III	17 (70.8)	10 (27.8)	27	
IVA	3 (12.5)	4 (11.1)	7	
Pathological grade				
G1	4 (16.7)	16 (44.4)	20	0.004
G2	10 (41.7)	17 (47.2)	27	
G3	10 (41.7)	3 (8.3)	13	

**Table 2:** Patient evaluation characteristics after neoadjuvant chemotherapy.

Characteristics	GASC1			*P* ^*∗*^
	High (*n* = 24, %)	Low (*n* = 36, %)	Total (*n* = 60)	
ypT				
T0	0	6 (16.7)	6	<0.001
Carcinoma in situ	0	3 (8.3)	3	
T1	1 (4.2)	7 (19.4)	8	
T2	2 (8.4)	9 (25)	11	
T3	10 (41.7)	8 (22.2)	18	
T4	11 (45.8)	3 (8.3)	14	
ypN				
N0	9 (37.5)	28 (77.8)	37	0.002
N^+^	15 (62.5)	8 (22.2)	23	
Pathological stage				
I	2 (8.3)	14 (38.9)	16	0.002
II	5 (20.8)	12 (33.3)	17	
III	13 (54.2)	10 (27.8)	23	
IVA	4 (16.7)	0	4	
Histologic grade				
G1	4 (16.7)	17 (47.2)	21	<0.001
G2	10 (41.7)	18 (50)	29	
G3	10 (41.7)	1 (2.8)	10	
Response				
CT evaluation				
CR	0	9 (25)	9	0.006
PR	8 (33.3)	16 (44.4)	24	
SD	13 (54.2)	11 (30.6)	24	
PD	3 (12.5)	0	3	
TRG				
1	0	6 (16.7)	6	0.006
2	5 (20.8)	15 (41.7)	20	
3	14 (58.3)	13 (36.1)	27	
4 + 5	5 (20.8)	2 (5.6)	7	
Surgical resection				
R0	18 (75)	34 (94.4)	52	0.046
R1	5 (20.8)	2 (5.6)	7	
R2	1 (4.2)	0	1	
TRG				
1	0	6 (16.7)	6	0.072
2–4	24 (100)	30 (83.3)	54	
ORR				
CR + PR	8 (33.3)	25 (69.4)	33	0.006
SD + PD	16 (66.7)	11 (30.6)	27	

**Table 3:** Univariate and multivariate analyses of risk factors for the overall survival.

	Univariate analysis	Multivariate analysis		
	*P* ^*∗*^	Hazard ratio	95% CI^*∗∗*^	*P* ^*∗∗∗*^
Age (year)				
<65/≥65	0.448			
Gender				
Male/female	0.679			
Tumor location				
Upper/middle/lower	0.156			
cT				
T1/T2/T3	0.028	2.366	1.233–4.569	0.01
cN				
N0/N+	0.014			
Pathological grade				
G1/G2/G3	0.044			
GASC1 expression				
High/low	0.039	1.89	1.011–4.216	0.048

^*∗*^Log-rank test. ^*∗∗*^Adjusted 95% confidence interval. ^*∗∗∗*^Cox proportional hazard model.

## Data Availability

The primary datasets used and/or analyzed during the current study are available from the corresponding author (Shegan Gao, gsg112258@haust.edu.cn) on reasonable request. The IRB and ethical committee of The First Affiliated Hospital of Henan University of Science will review the requests because of the patients' information.
